# Chronic Stress and Moral Decision-Making: An Exploration With the CNI Model

**DOI:** 10.3389/fpsyg.2018.01702

**Published:** 2018-09-11

**Authors:** Lisong Zhang, Ming Kong, Zhongquan Li, Xia Zhao, Liuping Gao

**Affiliations:** ^1^Institute of Disability Research, Nanjing Normal University of Special Education, Nanjing, China; ^2^School of Social and Behavior Sciences, Nanjing University, Nanjing, China; ^3^Department of Psychology, Soochow University, Suzhou, China

**Keywords:** chronic stress, deontology, moral judgment, omission bias, utilitarianism

## Abstract

Stress is prevalent in our daily life, and people often make moral decision-making in a stressful state. Several studies indicated the influence of acute stress on moral decision-making and behavior. The present study extended the investigation to chronic stress, and employed a new approach, the CNI model, to add new insights regarding the mechanism underlying the association between chronic stress and moral decision-making. A total of 197 undergraduates completed the Perceived Stress Scale and made moral decision-making on a series of deliberately designed moral dilemmas. The results indicated that higher chronic stress was related to more deontological moral choices. The process-dissociation analyses revealed that chronic stress was marginally significantly associated with deontological inclinations but not with utilitarian inclinations. And the CNI model analyses suggested that the high-stress group (above the median) showed a stronger general preference for inaction than the low-stress group (below the median) did, but there were no significant differences in sensitivity to consequences or sensitivity to moral norms between the two groups. Finally, the implications of the findings were discussed.

## Introduction

Stress is ubiquitous in current society, and people often make moral decisions under a stressful state (Singer et al., 2017). Even encountering moral scenarios induces stress experiences (Kälvemark et al., 2004). However, the direct investigation of how stress influences moral decision-making has not been conducted until the year 2011 (Starcke et al., 2011). Since then, the relationship between stress and moral decision-making has been gradually attracting researchers’ attention (Starcke et al., 2012; Youssef et al., 2012; Kossowska et al., 2016). The present study attempted to contribute to this field by exploring the association between chronic stress and moral judgment using a new approach, the CNI model (Gawronski et al., 2017).

Moral judgment is often referred to as evaluating the acceptability of an action or other characteristics on morality (Zhang et al., 2017a). The deontological vs. utilitarian underpinnings of moral judgment is an interesting topic in moral psychology. Hypothetical moral dilemmas have been widely used in these studies, and they help us understand the process (e.g., Cushman and Greene, 2012). Scholars traditionally considered only rationality was involved in the process (e.g., Kohlberg, 1971), while contemporary researchers emphasize both the roles of emotion and reason (e.g., Greene, 2007; Haidt, 2007). The dual-process theory of moral judgment proposed by Greene and his colleagues is among the well-known theories. According to this model, there are two independent processes involved in moral judgment: reason and emotion. Reasoning means that making a judgment mainly based on deliberation, while emotion implies that making a judgment mainly based on feelings. The final moral choices depend on the relative strength of the two processes. The theory has been supported by many empirical studies, both from behavioral and neuropsychological levels. Recently, some new theories have been proposed to explain findings in moral psychology, such as Graham et al. (2011) and Cushman (2013).

Although stress and moral judgment are closely related to each other in daily life, only a few studies have been conducted to explore their relationship so far. Some researchers found that stress led to more deontological judgments in traditional hypothetical moral dilemmas. Starcke et al. (2012) increased the stress level in the stress group with a cover-story of delivering a public speech, and asked participants to choose a utilitarian or a non-utilitarian alternative on 20 moral dilemmas. They found that fewer utilitarian judgments were made in the stress group than those in the control group while the average reaction time was longer for the stress group than that for the control group. In addition, increased physiological stress response indicated by heart rate was related to fewer utilitarian judgment. Youssef et al. (2012) increased the stress level in the stress group with the Trier Social Stress Test (TSST), and asked participants to make responses to three different types (non-moral, impersonal moral, and personal moral) of dilemmas with a total of thirty. Regarding the percentage of utilitarian responses, they found the stress group had significantly fewer utilitarian responses than the control group did on the personal dilemmas. However, Kossowska et al. (2016) found stress indicated by cortisol levels was not always related to deontological decisions, and the need for closure moderated the association between stress and moral decisions. Cortisol level was associated with more utilitarian judgment on in-group dilemmas (i.e., the Spelunkers dilemma and the Crying baby dilemma) for individuals with a high need for closure.

The previous studies have provided many fascinating insights into the relationship between stress and moral judgment. However, the drawbacks of the traditional dilemma approach they used might hinder further clarifying the psychological underpinnings of moral judgment (Gawronski et al., 2016). In their studies, accepting or rejecting a certain option (i.e., utilitarian action, or egocentric action), was regarded as endorsing either the deontological or utilitarian principle. There are two problems with the traditional approach: (1) The approach treated deontology and utilitarianism as bipolar opposites, while the dual process model suggested that the two moral principles were distinct and independent processes. (2) The approach conflated the utilitarian inclinations with the general tendency to action, and the deontological inclinations with the general tendency to inaction. Conway and Gawronski (2013) aimed to address the first problem, and proposed a process dissociation (PD) model to quantify the relative strength of utilitarian and deontological inclinations. Gawronski et al. (2017) further put forward a model with consequences, norms, and generalized inaction (CNI) to address both problems. They used multinominal modeling to provide parameter estimations of the three determinants of moral judgment: sensitivity to consequences, sensitivity to norms, and general tendency for inaction versus action without considering consequences and moral norms. According to Gawronski et al. (2016), the D parameter (deontological inclinations) in the PD model has a confound between sensitivity to moral norms and a general preference for inaction, while the U parameter (utilitarian inclinations) has a confound between sensitive to consequences (C parameter) and general preference for action. The CNI model has been applied to explore the effects of gender, cognitive load, question framing, psychopathy, and incidental emotions on moral judgment, and added new insights about the processes underlying these associations (Gawronski et al., 2017, 2018).

In addition, previous studies mainly focused on the effect of acute stress, and little is known about the association between chronic stress and moral decision-making. Stress could be distinguished as chronic stress and acute stress. Chronic stress is often referred to as the physiological reaction to stressors lasting for a quite extended period, while acute stress is the physiological reaction to specific challenging situations over a very short time (Caviola and Faulmüller, 2014). Compared to acute stress, chronic stress may have more profound impacts on the brain, cognition, and behavior (Lupien et al., 2009). Animal studies indicated that exposure to chronic stress resulted in more changes in prelimbic prefrontal cortex among rats than exposure to acute stress (Arnsten, 2009), and biased rats to quickly switch their behavioral strategies to habitual ones (Dias-Ferreira et al., 2009). Human studies revealed that chronic stress also found interacted with acute stress: acute stress impaired model-based control only among high chronic stressed participants (Radenbach et al., 2015). Moreover, chronic stress also interacted with cognitive speed on the model-based control (Friedel et al., 2017).

To address the gap, the present study aimed to extend the current investigation of stress and moral decision-making to chronic stress, and to provide new insights regarding the association using a CNI model. Existing studies demonstrated that chronic stress interfered with PFC function, inhibited cognitive control, and led to more habitual and intuitive responses (e.g., Arnsten, 2009; Dias-Ferreira et al., 2009). Based on the dual process model of moral judgment, we expected that higher chronic stress was related to more deontological judgment, and higher chronic stress group and lower chronic stress only have differences in deontological inclinations. According to the relationship between the PD approach and the CNI model approach, higher stress would be associated with increased sensitivity to moral norms, or enhanced general tendency of inaction, or both.

## Materials and Methods

### Participants

A total of 197 undergraduates (65.5% females and 34.4% males) were recruited among college students enrolled in a psychology course. Their age ranged from 18 to 22 years old (*M* = 19.49, *SD* = 0.83), and most of them were sophomores (83.8%). In addition, most of them majored in psychology (72.1%).

### Measures

#### Moral Dilemmas

A set of 24 scenarios, four parallel forms of six fundamental moral dilemmas, were adopted from Gawronski et al. (2017). The parallel forms were constructed by varying two factors: norms (proscriptive norm that prohibits action versus prescriptive norm that prescribes action) and consequences (benefits of action greater than its costs versus benefits of action smaller than its costs). Each of the dilemmas described a moral scenario where the moral agent had to decide on whether to perform a specific action. Dilemmas were presented to participants one by one on the screen in a fixed random order. Participants first read the description of each moral dilemma, then make a judgment on the acceptability of a particular action, yes or no.

#### The Perceived Stress Scale

The scale was used to assess individual’s subjective perceived chronic stress, and it is among the most widely used instruments for this purpose (Wang et al., 2011). The 14-item version of the scale was first developed by Cohen and his colleagues (Cohen et al., 1983), and later the 10-item version demonstrated with sound psychometric properties (Cohen and Williamson, 1988). A Chinese revision of the 10-item version scale was used in the present study (Wang et al., 2011). Participants indicated how often they felt or thought a certain way as described by each item during the last month on a five-point Likert scale from 0 (never) to 4 (very often). The scores on the ten items are summed up to calculate a global index for chronic stress, which represented a global (i.e., domain-general) and chronic (i.e., across the past month) level of subjectively experienced stress (Cohen et al., 1983). The reliability computed as Cronbach’s α coefficient was 0.80 in the current sample.

### Data Analysis

For the moral dilemmas, the choice of “yes” (accepting a choice of action) was recorded as 1 and the choice of “no” (unaccepting a choice of action) was recorded as 0. In the traditional analyses, an index of the percentage of utilitarian options was treated as the dependent variable. In the process-dissociation analysis, utilitarian inclinations and deontological inclinations were qualified and treated as dependent variables. And in the CNI model analyses, comparisons of model fit and parameters were performed. The software multiTree (Moshagen, 2010) and the multiTree template file for CNI model analyses (Gawronski et al., 2017) were used in the CNI model analyses, while SPSS 22.0 was used in all other analyses.

## Results

**Table 1** presents the descriptive statistics of major variables, including the mean, standard deviation, and correlations. From the **Table 1**, we can see there was a significant negative correlation between scores on the PSS and moral judgment, *r* = −0.16, *p* = 0.022. That is, higher chronic stress was related to lower rating on moral appropriateness of the utilitarian actions. In addition, scores on the PSS had a significant positive correlation with the D parameters, *r* = 0.14, *p* = 0.045. However, the correlation between scores on the PSS and the U parameter did not reach statistical significance at 0.05 levels, *r* = −0.027, *p* = 0.703.

**Table 1:** Means, standard deviation, and correlations for major variables.

Variables	*M*	*SD*	1	2	3	4	5
(1) Age	19.49	0.83					
(2) Gender	0.35	0.48	0.08				
(3) Stress	26.51	5.33	0.05	−0.12			
(4) Moral choice	2.79	0.95	−0.13	0.01	−0.16*		
(5) U parameter	0.17	0.23	0.00	−0.05	−0.03	0.17*	
(6) D parameter	0.52	0.24	0.12	−0.11	0.14*	−0.70**	0.32^∗∗^

*Note: N = 197. Gender: 0 = female, 1 = male. ^∗^p < 0.05, ^∗∗^p < 0.01.*

### Traditional Analyses

The traditional moral dilemma approach only considered one form of moral dilemmas in the set, dilemmas in which a proscriptive norm prohibits action, and the benefits of an action outweighs its costs for overall well-being. On this type of dilemmas, accepting a choice of action would be interpreted as a preference for utilitarian response, and unaccepting a choice of action would be interpreted as a preference for deontological responses. We averaged the choices across the six dilemmas of this type, and found in general participants showed a slight preference for deontological responses over utilitarian responses (*M* = 2.79, *SD* = 0.95). Further analysis indicated that the overall preference score was significantly different from the neutral reference point of 3, *t* (196) = 3.06, *p* < 0.01, d = 0.22.

Previous studies have indicated age and gender differences in moral judgment. Females express less preference for utilitarian options than males, while the older made less utilitarian judgments than the younger (e.g., Arutyunova et al., 2016). Friesdorf et al. (2015) found that females had higher deontological inclinations than males. Armstrong et al. (in press) further revealed that gender differences in deontological inclinations were resulted from harm aversion and action aversion. We also examined whether scores on the PSS predicted responses on moral dilemmas using hierarchical multiple regression analysis. In the model, the average of utilitarian moral choices was treated as the dependent variable, and age and gender were entered into the first block as control variables, and stress into the second block (see **Table 2**). The result indicated that after controlling the effect of age and gender, chronic stress could still significantly predict moral dilemma judgments, *β* = −0.16, *p* = 0.03. Higher chronic stress was related to less endorsement of utilitarian actions.

**Table 2:** Summary of hierarchical multiple regression analyses for predicting utilitarian moral judgment.

Variables	Model 1	Model 2
Age	−0.13	−0.12
Gender	0.02	0.01
Stress		−0.16*
R^2^	0.02	0.04
ΔR^2^		0.02*

*Note: N = 197. ^∗^p < 0.05. The standardized regression coefficients are presented.*

### Process-Dissociation Analyses

According to the procedure proposed by Conway and Gawronski (2013), we calculated the process-dissociation parameters for each participant. In the current study, moral dilemmas with proscriptive norms involving benefits of action that are greater than the cost of action were treated as incongruent dilemmas, and moral dilemmas with proscriptive norms involving benefits of action that are smaller than the cost of action were treated as congruent dilemmas. PD utilitarian and deontological parameters were standardized before the following analysis.

We further examined whether scores on the PSS predicted utilitarian and deontological inclinations separately. In the regression models, the U parameter or the D parameter was treated as the dependent variable, and age and gender were entered into the first block as control variables, and stress into the second block (see **Table 3**). The result indicated that after controlling the effect of age and gender, chronic stress had a marginally significant association with deontological inclinations, *β* = 0.13, *p* = 0.08, while chronic stress could not significantly predict utilitarian inclinations, *β* = −0.04, *p* = 0.63. In a word, chronic stress was associated with deontological inclinations but not with utilitarian inclinations.

**Table 3:** Summary of hierarchical multiple regression analyses for predicting deontological and utilitarian inclinations.

Variables	Deontological inclinations		Utilitarian inclinations	
	Model 1	Model 2	Model 1	Model 2
Age	0.13^#^	0.12^#^	0.004	0.006
Gender	−0.13^#^	−0.11	−0.06	−0.06
Stress		0.13^#^		−0.04
*R^2^*	0.03	0.05	0.003	0.004
Δ*R^2^*		0.02^#^		0.001

*Note: N = 197. #p < 0.10. The standardized regression coefficients are presented.*

### CNI Model Analyses

The CNI model analysis requires categorical independent variables for model and parameter comparisons (Gawronski et al., 2017), and the median-split method is recommended for dichotomizing a continuous variable (Iacobucci et al., 2015). To facilitate the CNI model analyses, we first divided participants into two groups in terms of their scores on the Perceived Stress Scale, then compared the different performance of the high- and low-stress groups. Participants with scores above the median were assigned to the high-stress group (*M* = 31.14, *SD* = 3.28, *N* = 92), while participants with scores below the median were assigned to the low-stress group (*M* = 22.45, *SD* = 2.91, *N* = 105).

We analyzed the data as a whole, and the CNI model fit the data well, *G*^2^ (1) = 2.44, *p* = 0.12. The C parameter (*M* = 0.131, 95% CI [0.102, 0.159]) was significantly larger than zero, Δ*G*^2^ (1) = 80.46, *p* < 0.001, indicating that participants were highly sensitive to consequences in moral decision-making. The N parameter (*M* = 0.145, 95% CI [0.113, 0.178]) significantly deviated from zero, Δ*G*^2^(1) = 75.17, *p* < 0.001, implying that participants were highly sensitive to norms in moral decision-making. There was a significant difference between the I parameter (*M* = 0.451, 95% CI [0.431, 0.470]) and its neutral reference point of 0.5, Δ*G*^2^(1) = 25.18, *p* < 0.001, indicating that participants expressed a higher preference for action over inaction.

**FIGURE 1:**
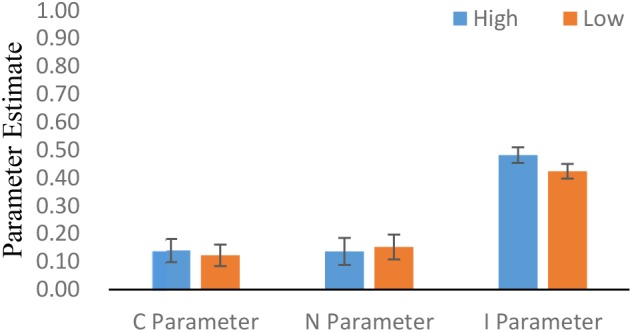
Estimates of three parameters for both groups: sensitivity to consequences (*C*), sensitivity to norms (*N*), and a general preference for inaction versus action (*I*). Error bars reflect 95% confidence intervals.

We also analyzed the data separately for participants in the high-stress group and the low-stress group, and the CNI model still fit the data well, *G^2^*(2) = 2.89, *p* = 0.24. The high-stress group and the low-stress group had no significant difference on the C parameter, Δ*G^2^*(1) = 0.363, *p* = 0.547, and on the N parameter, Δ*G*^2^(1) = 0.218, *p* = 0.641. However, participants in the high-stress group showed significantly higher scores on the I parameter than those in the low-stress group, Δ*G*^2^(1) = 8.718, *p* = 0.003. That is, participants in the high-stress group showed a stronger general preference for inaction compared with those in the low-stress group (see **Figure 1**).

## Discussion

The present study extended the investigation the relationship between stress and moral judgment from acute stress to chronic stress using a CNI model. Participants were faced with moral dilemmas in which action was prohibited by a proscriptive moral norm even though the consequences of action would produce more overall well-being. When judging dilemmas like these, chronic stress was negatively associated with a willingness to engage in such action. The PD analyses revealed that higher chronic stress was marginally significantly associated with increased deontological inclinations but not with utilitarian inclinations. In addition, the CNI model analyses indicated that the difference between participants with high stress and with low stress was driven by a general preference for inaction versus action regardless of consequences and norms. Those findings were consistent with our hypotheses.

Traditional analyses indicated that higher stress was associated with more deontological moral choices. It was consistent with the findings of some studies on acute stress and moral decision-making. Both Starcke et al. (2012) and Youssef et al. (2012) found stressed participants made less utilitarian responses than their counterparts in the control group. Stress triggers a shift from analytical reasoning to intuitive processes, and resulted in more spontaneous and innate responses (Yu, 2016). Regarding moral decision-making, more reliance on intuitive responses would lead to more deontological choices in terms of the dual process model (Greene, 2007). However, Kossowska et al. (2016) reported the main effect of cortisol level was not significant, but for a special group participants with a high need for closure, higher cortisol levels were related to more utilitarian judgments on in-group dilemmas. This divergence might be due to the differences in their operations of stress and the types of dilemmas they focused.

Stress was related to deontological moral decisions, but it was still not clear whether the result was caused by increased deontological inclinations or decreased utilitarian inclinations, or both (Conway and Gawronski, 2013). The results of PD analyses shed new light on the roles of deontological and utilitarian inclinations in the association between stress and moral decision-making. Higher stress was selectively related to increased deontological inclinations, and the association reached marginal significance. The finding was in agreement with the above explanation on the basis of the stress induced deliberation-to-intuition (SIDI) model (Yu, 2016) and the dual process model of moral decision-making (Greene, 2007). Stress results in the dominance of fast, automatic and emotional intuitions (Yu, 2016). Deontological inclinations and utilitarian inclinations are rooted in emotional reactions to harmful action, and deliberative analyses on the cost-benefit of the outcome of adverse action (Greene, 2007; Conway and Gawronski, 2013; Zhang et al., 2017b). Therefore, stress was associated with deontological inclinations, but had nothing to do with utilitarian inclinations. While the marginally significant association between chronic stress and deontological inclinations is intriguing for future research, we do not have sufficient justification to reject the null hypothesis with a single sample.

The CNI model analyses further clarified the potential confound between the general preference for inaction with sensitivity to moral norms in the deontological inclination parameter estimation, and between the general preference for action with sensitivity to consequences in the utilitarian inclination parameter estimation (Gawronski et al., 2017). Our results indicated that compared to the low-stress group, the high-stress group had similar sensitivity to norms and consequences, but had a higher general preference for inaction. That is, higher stress was related to increased general preference to not to engage in any action. Stress impairs individuals’ cognitive control (Hermans et al., 2017), diminishes their confidence in making an optimal decision, and reduces their willingness to engage in the action (Gawronski et al., 2017). Typically for the same amount of harm, the action caused one is considered worse than that caused by inaction, which is called omission bias (Cushman et al., 2006).

However, some limitations should be mentioned. A causal inference about the relationship between chronic stress and moral decision-making could not be made due to the current correlational design. It is not ethical and realistic to induce chronic stress among human participants. However, examining the moral choices of some patient groups, such as people with depression and post-traumatic stress disorder, would shed new light on this relationship (Starcke and Brand, 2012). Moreover, there are some potential confounds in the association between chronic stress and moral dilemma judgment, such as feelings of lack of control and mental health level. Learned helplessness also provided a possible explanation for the association between chronic stress and the general preference for inaction in moral judgment. Chronic stress leads to learned helplessness (Katz et al., 1981), while inaction tendency is a feature of learned helplessness (Overmier, 2002). Further studies are needed to clarify the potential confounds. In addition, only choice responses were collected in the present study. Response time and biological indexes (such as cortisol level) should be included to provide a more comprehensive understanding (Youssef et al., 2012).

In sum, the present study employed a new approach, the CNI model, to investigate the relationship between chronic stress and moral decision-making. Our findings indicated that higher stress was associated with an enhanced general preference for inaction, and increased deontological choices. It provided novel insights into the underlying mechanisms.

## Ethics Statement

This study was carried out in accordance with the recommendations of Ethical Conduct in Human Research by the Institutional Review Board of Department of Psychology, Nanjing University, with written informed consent from all subjects. All subjects gave written informed consent in accordance with the Declaration of Helsinki. The protocol was approved by the Institutional Review Board of Department of Psychology, Nanjing University.

## Author Contributions

All authors conceived, designed, and conducted the studies. ZL conducted the statistical analyses. LZ and MK wrote the first draft and all authors revised the final manuscript.

## Conflict of Interest Statement

The authors declare that the research was conducted in the absence of any commercial or financial relationships that could be construed as a potential conflict of interest.

## References

[B1] Armstrong J. Friesdorf R. Conway P. (in press). Clarifying gender differences in moral dilemma judgments: the complementary roles of harm aversion and action aversion. *Soc. Psychol. Personal. Sci.* 10.1177/1948550618755873

[B2] Arnsten A. F. T. (2009). Stress signalling pathways that impair prefrontal cortex structure and function. *Nat. Rev. Neurosci.* 10 410–422. 10.1038/nrn2648 19455173 PMC2907136

[B3] Arutyunova K. R. Alexandrov Y. I. Hauser M. D. (2016). Sociocultural influences on moral judgments: East–West, male–female, and young–old. *Front. Psychol.* 7:1334. 10.3389/fpsyg.2016.01334 27656155 PMC5011137

[B4] Caviola L. Faulmüller N. (2014). “How stress influences our morality,” in *Mind Magazine*, ed. Laysee O. (Oxford: University of Oxford), 1–6.

[B5] Cohen S. Kamarck T. Mermelstein R. J. (1983). A global measure of perceived stress. *J. Health Soc. Behav.* 24 385–396. 10.2307/2136404 6668417

[B6] Cohen S. Williamson G. (1988). “Perceived stress in a probability sample of the United States,” in *The Social Psychology of Health: Claremont Symposium on Applied Social Psychology*, eds Spacapan S. Oskamp S. (Newbury Park, CA: Sage), 31–67.

[B7] Conway P. Gawronski B. (2013). Deontological and utilitarian inclinations in moral decision making: a process dissociation approach. *J. Pers. Soc. Psychol.* 104 216–235. 10.1037/a0031021 23276267

[B8] Cushman F. (2013). Action, outcome, and value: a dual-system framework for morality. *Pers. Soc. Psychol. Rev.* 17 273–292. 10.1177/1088868313495594 23861355

[B9] Cushman F. Young L. Hauser M. (2006). The role of conscious reasoning and intuition in moral judgment: testing three principles of harm. *Psychol. Sci.* 17 1082–1089. 10.1111/j.1467-9280.2006.01834.x 17201791

[B10] Cushman F. A. Greene J. D. (2012). Finding faults: how moral dilemmas illuminate cognitive structure. *Soc. Neurosci.* 7 269–279. 10.1080/17470919.2011.614000 21942995

[B11] Dias-Ferreira E. Sousa J. C. Melo I. Morgado P. Mesquita A. R. Cerqueira J. J. (2009). Chronic stress causes frontostriatal reorganization and affects decision-making. *Science* 325 621–625. 10.1126/science.1171203 19644122

[B12] Friedel E. Sebold M. Kuitunen-Paul S. Nebe S. Veer I. M. Zimmermann U. S. (2017). How accumulated real life stress experience and cognitive speed interact on decision-making processes. *Front. Hum. Neurosci.* 11:302. 10.3389/fnhum.2017.00302 28642696 PMC5462964

[B13] Friesdorf R. Conway P. Gawronski B. (2015). Gender differences in responses to moral dilemmas: a process dissociation analysis. *Pers. Soc. Psychol. Bull.* 42 696–713. 10.1177/0146167215575731 25840987

[B14] Gawronski B. Armstrong J. Conway P. Friesdorf R. Hütter M. (2017). Consequences, norms, and generalized inaction in moral dilemmas: the CNI model of moral decision-making. *J. Pers. Soc. Psychol.* 113 343–376. 10.1037/pspa0000086 28816493

[B15] Gawronski B. Conway P. Armstrong J. Friesdorf R. Hütter M. (2016). “Understanding responses to moral dilemmas,” in *The Social Psychology of Morality*, eds Forgas J. P. Jussim L. Lange P. A. M. V. (New York, NY: Psychology Press), 91–110.

[B16] Gawronski B. Conway P. Armstrong J. Hütter M. (2018). Effects of incidental emotions on moral dilemma judgments: an analysis using the CNI model. *Emotion* 10.1037/emo0000399 [Epub ahead of print]. 29389208

[B17] Graham J. Nosek B. A. Haidt J. Iyer R. Koleva S. Ditto P. H. (2011). Mapping the moral domain. *J. Pers. Soc. Psychol.* 101 366–385. 10.1037/a0021847 21244182 PMC3116962

[B18] Greene J. (2007). Why are VMPFC patients more utilitarian? A dual-process theory of moral judgment explains. *Trends Cogn. Sci.* 11 322–323. 10.1016/j.tics.2007.06.004 17625951

[B19] Haidt J. (2007). The new synthesis in moral psychology. *Science* 316 998–1002. 10.1126/science.1137651 17510357

[B20] Hermans E. J. Henckens M. J. A. G. Joëls M. Fernández G. (2017). “Time-dependent shifts in neural systems supporting decision-making under stress,” in *Decision Neuroscience*, eds Dreher J. Tremblay L. (San Diego, CA: Academic Press), 371–385.

[B21] Iacobucci D. Posavac S. S. Kardes F. R. Schneider M. J. Popovich D. L. (2015). The median split: robust, refined, and revived. *J. Consum. Psychol.* 25 690–704. 10.1016/j.jcps.2015.06.014

[B22] Kälvemark S. Höglund A. T. Hansson M. G. Westerholm P. Arnetz B. (2004). Living with conflicts-ethical dilemmas and moral distress in the health care system. *Soc. Sci. Med.* 58 1075–1084. 10.1016/S0277-9536(03)00279-X 14723903

[B23] Katz R. J. Roth K. A. Carroll B. J. (1981). Acute and chronic stress effects on open field activity in the rat: implications for a model of depression. *Neurosci. Biobehav. Rev.* 5 247–251. 10.1016/0149-7634(81)90005-1 7196554

[B24] Kohlberg L. (1971). “From is to ought: How to commit the naturalistic fallacy and get away with it in the study of moral development,” in *Cognitive Development and Epistemology*, ed. Mischel T. (New York, NY: Academic Press), 151–235.

[B25] Kossowska M. Czernatowicz-Kukuczka A. Szumowska E. Czarna A. (2016). Cortisol and moral decisions among young men: the moderating role of motivation toward closure. *Pers. Individ. Differ.* 101 249–253. 10.1016/j.paid.2016.06.017

[B26] Lupien S. J. McEwen B. S. Gunnar M. R. Heim C. (2009). Effects of stress throughout the lifespan on the brain, behaviour and cognition. *Nat. Rev. Neurosci.* 10 434–445. 10.1038/nrn2639 19401723

[B27] Moshagen M. (2010). multiTree: a computer program for the analysis of multinomial processing tree models. *Behav. Res. Methods* 42 42–54. 10.3758/brm.42.1.42 20160285

[B28] Overmier J. B. (2002). On learned helplessness. *Integr. Physiol. Behav. Sci.* 37 4–8. 10.1007/BF02688801 12069364

[B29] Radenbach C. Reiter A. M. F. Engert V. Sjoerds Z. Villringer A. Heinze H.-J. (2015). The interaction of acute and chronic stress impairs model-based behavioral control. *Psychoneuroendocrinology* 53 268–280. 10.1016/j.psyneuen.2014.12.017 25662093

[B30] Singer N. Sommer M. Döhnel K. Zänkert S. Wüst S. Kudielka B. M. (2017). Acute psychosocial stress and everyday moral decision-making in young healthy men: the impact of cortisol. *Horm. Behav.* 93 72–81. 10.1016/j.yhbeh.2017.05.002 28495558

[B31] Starcke K. Brand M. (2012). Decision making under stress: a selective review. *Neurosci. Biobehav. Rev.* 36 1228–1248. 10.1016/j.neubiorev.2012.02.003 22342781

[B32] Starcke K. Ludwig A.-C. Brand M. (2012). Anticipatory stress interferes with utilitarian moral judgment. *Judgm. Decis. Mak.* 7 61–69.

[B33] Starcke K. Polzer C. Wolf O. T. Brand M. (2011). Does stress alter everyday moral decision-making? *Psychoneuroendocrinology* 36 210–219. 10.1016/j.psyneuen.2010.07.010 20692104

[B34] Wang Z. Chen J. Boyd J. E. Zhang H. Jia X. Qiu J. (2011). Psychometric properties of the Chinese version of the perceived stress scale in policewomen. *PLoS One* 6:e28610. 10.1371/journal.pone.0028610 22164311 PMC3229602

[B35] Youssef F. F. Dookeeram K. Basdeo V. Francis E. Doman M. Mamed D. (2012). Stress alters personal moral decision making. *Psychoneuroendocrinology* 37 491–498. 10.1016/j.psyneuen.2011.07.017 21899956

[B36] Yu R. (2016). Stress potentiates decision biases: a stress induced deliberation-to-intuition (SIDI) model. *Neurobiol. Stress* 3 83–95. 10.1016/j.ynstr.2015.12.006 27981181 PMC5146206

[B37] Zhang L. Kong M. Li Z. (2017a). Emotion regulation difficulties and moral judgment in different domains: the mediation of emotional valence and arousal. *Pers. Individ. Differ.* 109 56–60. 10.1016/j.paid.2016.12.049

[B38] Zhang L. Li Z. Wu X. Zhang Z. (2017b). Why people with more emotion regulation difficulties made a more deontological judgment: the role of deontological inclinations. *Front. Psychol.* 8:2095. 10.3389/fpsyg.2017.02095 29234299 PMC5712370

